# A review of robotic and automated systems in meat processing

**DOI:** 10.3389/frobt.2025.1578318

**Published:** 2025-05-23

**Authors:** Yining Lyu, Fan Wu, Qingyu Wang, Guanyu Liu, Yingqi Zhang, Huanyu Jiang, Mingchuan Zhou

**Affiliations:** ^1^ College of Biosystems Engineering and Food Science, Zhejiang University, Hangzhou, China; ^2^ Key Laboratory of Intelligent Equipment and Robotics for Agriculture of Zhejiang Province, Zhejiang University, Hangzhou, China; ^3^ Chair of Robotics, Artificial Intelligence and Real-time Systems, Technische Universität München, München, Germany

**Keywords:** meat processing system, automated equipment, meat production automation, meat processing robot, robot processing system

## Abstract

Tasks in the meat processing sector are physically challenging, repetitive, and prone to worker scarcity. Therefore, the imperative adoption of mechanization and automation within the domain of meat processing is underscored by its key role in mitigating labor-intensive processes while concurrently enhancing productivity, safety, and operator wellbeing. This review paper gives an overview of the current research for robotic and automated systems in meat processing. The modules of a robotic system are introduced and afterward, the robotic tasks are divided into three sections with the features of processing targets including livestock, poultry, and seafood. Furthermore, we analyze the technical details of whole meat processing, including skinning, gutting, abdomen cutting, and half-carcass cutting, and discuss these systems in performance and industrial feasibility. The review also refers to some commercialized products for automation in the meat processing industry. Finally, we conclude the review and discuss potential challenges for further robotization and automation in meat processing.

## 1 Introduction

Meat consumption is widely acknowledged as an indispensable source of vital nutrition for individuals worldwide. Technician’s market research report indicates the meat market is for substantial expansion, with a projected value reaching USD 1210.97 billion by 2027. During the forecast period, an anticipated compound annual growth rate of approximately 7% is foreseen (Technavio, 2023). To address the heightened demand for meat, an estimated 80 billion animals undergo slaughter annually, which has significantly increased the meat processing workload (Ritchie, 2017). This accelerated growth motivated the meat industry’s increasing need for heightened efficiency and productivity, which has expedited the automation market’s growth within the entire meat industry.

Meat processing poses safety and contamination risks (Hamid et al., 2016; Das et al., 2019). There is a strong demand for automation to solve these problems, but it is still challenging in the whole meat process automation due to the high product variability (Romanov et al., 2022), dexterous manipulation, harsh environment, and space constraints (Singh et al., 2012).

Compared with other industries, the meat industry’s working environment is not very conducive to robotics. Meat processing automation is constrained by equipment sensitivity to size variations (Barbut, 2014) and material deformability, necessitating adaptive robotics. The depiction of meat distinctiveness and diversity, together with the mechanization and automation of processes, poses a formidable trial for Meat Industry 4.0. The initiative’s overarching objective is to secure superior quality, safety, and traceability in the meat sector through the application of advanced industry 4.0 technologies such as artificial intelligence, big data, robotics, smart sensors, and blockchain (Echegaray et al., 2022; Wang et al., 2024).

Due to the complexity of meat processing, which requires balancing efficiency with operational precision, meat processing automation faces several inherent challenges. Meat, being a typical non-standard flexible material, imposes high demands on robotic manipulation. The variation in the mechanical properties of different meat parts, such as its viscoelasticity, necessitates sophisticated control systems that can adapt to these inconsistencies. For instance, the automation system should manage multi-module coordination, integrating path planning for cutting with real-time force control. The system must be capable of adjusting forces dynamically based on the varying elasticity and stickiness of the meat at different points, ensuring both precision and safety. Moreover, when processing large livestock such as pigs, cows, and sheep, the scale of the equipment required becomes even more significant. These larger animals demand heavier and more robust machinery, which also has to accommodate the increased range of motion necessary for efficient processing. Robotic arms, in particular, must possess greater degrees of freedom to effectively plan and execute complex movements during the cutting and processing stages.

**FIGURE 1:**
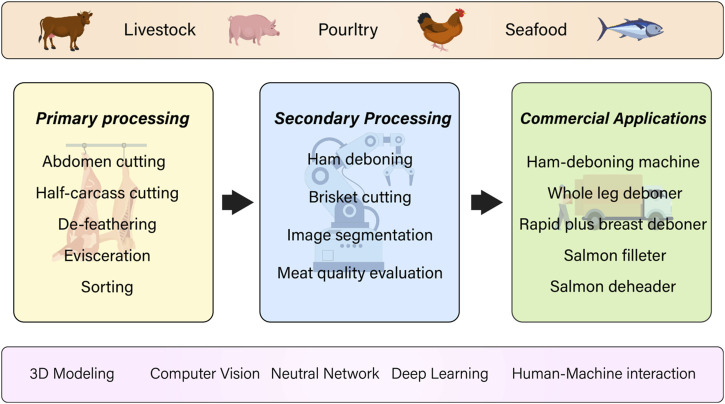
Flowchart of the technology demonstrated in this review.

In this review, a comprehensive summary of recent progress in automatic and intelligent meat processing systems is presented, as depicted in Figure 1. The organization of the paper is structured as follows: In Section 2, a concise overview is provided of the key modules that make up a robotic system for meat processing. Additionally, a comprehensive analysis of relevant literature on this subject matter will be provided. The subsequent three sections are dedicated to discussing specific robotic tasks involved in meat processing, focusing on livestock (Section 3), poultry (Section 4), and seafood (Section 5) respectively. Section 6 delves into recent research trends in meat processing and analysis, presenting insights from recent studies. Finally, Section 7 brings the paper to a conclusion by summarizing the main findings and outlining future directions for research in this area.

## 2 Methodology

In principle, a meat-processing robotic system requires three major modules including a sensing and perception module, a control module, and an actuation module. In meat processing, sensing plays a crucial role in ensuring accurate execution. Effectively utilizing pertinent sensor information constitutes a pivotal factor in expanding the system’s functionality. As per the varying sensing requirements, the tasks to be performed before meat processing can be segregated into three primary categories: external features, internal features, and tissue boundaries. There has been some research on the practical application of different sensing systems including beef ear tag detection using color cameras (Kumar et al., 2017), pig organ grasping with force and torque sensors (Takács et al., 2021), laser profiling for sheep head removal (Condie et al., 2007), the feather bone detection along the beef carcass spine.

In meat processing, the main usage of the control component is producing cutting routes by utilizing the extracted outline of the animal body. The path derived from the contour may be highly intricate (Meng et al., 2024). Hence, the process involves discretizing it into multiple points, followed by fitting a seamless curve through these points. Additionally, the control module involves the alignment process among the robot tool coordinate systems (TCS), user coordinate systems (UCS), and camera coordinate systems (CCS).

The actuation module is deployed to execute the predefined tasks along the trajectory generated by the control module. Typically, the terminal actuator employed in meat processing is equipped with highly specialized functionalities. Robotic manipulators provided by reputable companies such as Motoman, KUKA, FANUC, and ABB are commonly employed as actuators. Robotic hands exhibit a versatile capacity to perform a diverse array of tasks facilitated by a variety of end manipulators.

Thus far, within the agri-food sector, scholars have provided valuable insights regarding the implementation of meat processing robots. In this study, we analyze selected academic papers. Furthermore, the interdependent relationship between equipment development automation and intelligent technologies is also obvious.

## 3 Livestock

Livestock production is a vital component of sustainable food systems. It provides animal protein crucial for proper health, implements environmentally sustainable production methods prioritizing conservation efforts, and fosters the growth of rural communities worldwide. The livestock industry holds a unique position as a leader and substantial contributor to global food system discussions. As the human population and incomes increase, there has been a notable increase in the volume of meat production on a global scale (Godfray et al., 2018; Liu et al., 2023). It is anticipated that by the year 2029, the aggregate growth rate of red meat production will soar by 80% (Schmidhuber et al., 2020). Facing this situation, extending meat processing automation is imperative.

Modern livestock meat processing can be divided into several main steps: livestock handling, primary processing, secondary processing, packaging, and labeling (Esper et al., 2021). In Table 1, recent research on the identification and classification of livestock has been listed. In this section, we provide an overview of the recent advancements in automation within the meat processing industry. This includes abdomen cutting and half carcass cutting in primary processing, and ham deboning, brisket cutting, and carcass image segmentation in secondary processing. Furthermore, current commercialized processing machines and some quality assessment techniques for pork, beef, and mutton are also mentioned.

**TABLE 1:** Summary of automatic and robotic cutting of livestock segment processing.

References	Country	Technique	Purpose	Hardware	Software and algorithms	Livestock
Fortin et al. (2003)	Canada	Machine vision Ultrasound imaging system	Pork carcass grading	Aloka SSD 1100 ultrasound machine Charge-coupled device camera	Computer vision system	Pork
Condie et al. (2007)	Australia	Machine vision	Sheep brisket cutting	CV-3200 camera	Prototype automated system	Sheep
Liu et al. (2017)	China	Machine vision	Porcine abdomen cutting	2D camera	Global optimization algorithm Genetic algorithm	Pork
Mu et al. (2020)	China	Machine vision	Half-Sheep cutting	Kinect camera	Deeplab v3+ networks	Sheep
Cong et al. (2019)	China	Machine vision	Porcine abdomen cutting	Binocular camera	Image recognition algorithm	Pork
Cong et al. (2021)	China	Machine vision	Porcine abdomen cutting	Individual RGB-D camera	Kernel principal component analysis Binocular vision techniques	Pork
Bao et al. (2022)	China	Machine vision	Sheep carcass cutting	Azure Kinect camera	-	Sheep

### 3.1 Primary processing of livestock

Primary livestock processing is an extremely crucial step that demands high hygiene, quality, and accuracy standards to ensure proper subsequent operations (Cong et al., 2021). It concerns activities within a slaughterhouse including stunning, dressing, viscera removing abdomen cutting, and so on (Longdell, 1996).

**FIGURE 2:**
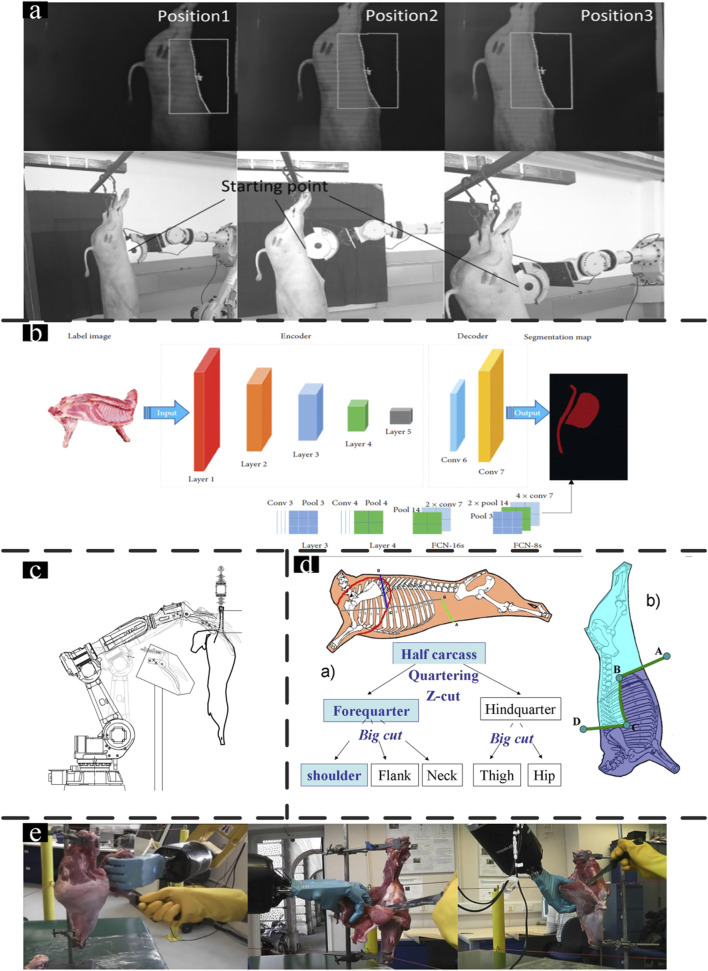
Livestock processing robot: **(a)** Feature identification with the profile moved (Liu et al., 2017); **(b)** FCN image segmentation model (Mu et al., 2020); **(c)** The axis representation for carcass in operational space (Singh et al., 2012); **(d)** Beef preparation process and Z-cut (Guire et al., 2010a); **(E)** Metamorphic hand integrated with ABB robot (Wei et al., 2014).

With the increasing application of machine vision related to meat analysis and livestock identification, vision-based robots have been studied. Based on a genetic algorithm, Liu et al. (2017) proposed a flexible robotic cutting system based on a genetic algorithm that utilizes trajectory planning a 2D camera captures the pig’s side view, and MATLAB extracts the abdominal curve, which is fitted with a fifth-order spline. The path is then optimized using a genetic algorithm (GA), minimizing cutting segments and errors. The optimized path is discretized into six segments, with a maximum cutting error of 1.6 mm, ensuring accurate cuts through the skin and muscle while avoiding internal organ damage. However, the recognized accuracy is still low. The abdominal cutting curve obtained through the image binarization shows limited alignment with the actual carcass, resulting in constrained cutting success rates. (Figure 2a) (Esper et al., 2021). Computer version techniques such as deep learning and 3D modeling could provide higher accuracy compared to machine vision (Kang et al., 2022), they bring more and better opportunities to meat processing systems. Deeplab v3+ networks used for a half-sheep cutting Robotic 3D Vision-Guided System acquire the key cutting points and the system stability is acceptable. As is one of the most complicated operations in meat processing. The result indicates the automation feasibility even for the hardest step in meat production and indicates the application potential for 3D versions in carcass cutting (Mu et al., 2020).

The 3D vision system is a kind of method with the ability to record the 3D features of the carcass in both coordinates and vectors. Grounding on the New Zealand sheep body segmentation specification, Mu et al. (2020) developed a segmentation robot for half-sheep cutting (Figure 2b). The 3D camera was used to obtain the depth image of the sheep carcass, and the deep image processing algorithms were employed to acquire the key cutting points. The cutting robot trajectory is planned according to the spatial coordinates of processed point clouds. While the system excels in cutting path planning, it still relies on open-loop control and does not incorporate force sensors for feedback, limiting its ability to detect sudden changes in cutting resistance. Additionally, the pre-defined cutting paths may struggle to adapt to the variability in carcass structure, posing a challenge to the system’s overall flexibility and precision. Another method of cutting sheep carcasses based on a 3D vision system with a dual-robot system was proposed by Bao et al. (2022). Compared to traditional robotics systems, the dual-robot system has the advantage of large operating space and is suitable for sheep carcass processing. A fixed device is designed to prevent the carcass swing in the cutting process, and the dual-robot system performs precision cutting according to the trajectory calculated by the point cloud spatial coordinates. These methods have shown promising results that can improve processing efficiency, accuracy, and consistency while reducing labor costs and improving worker safety.

The reviewed vision-based robotic systems demonstrate progressive advancements in meat processing automation. While 2D machine vision (Liu et al., 2017) struggles with alignment accuracy (70%–80% success), 3D vision systems (Mu et al., 2020; Bao et al., 2022) achieve superior precision (85%–92%) through point-cloud spatial mapping and dual-robot coordination. Deep learning-enhanced methods (Kang et al., 2022) further improve robustness against anatomical variability but require higher computational resources. Collectively, 3D vision and AI integration represent the most viable path toward fully automated, high-throughput carcass processing, though cost-effectiveness remains a challenge for small-scale operations (Liu et al., 2024).

### 3.2 Secondary processing of livestock

Secondary processing mainly involved primal brisket cutting and deboning. These tasks currently are performed manually other than automation due to the challenges of complicated manipulation and soft tissue characteristics. Examining manual tasks aids in gaining a comprehensive understanding of the prototype specifications. Brisket cutting is the operation after the processing of the viscera. Hence, the brisket-cutting process demands a heightened level of precision, labor expertise, and uniformity to ensure the attainment of superior meat quality (Singh et al., 2012).

By investigating the manual task of brisket cutting, Condie et al. (2007) developed a brisket-cutting robot with a laser profile analysis system. The sensing system, comprising image acquisition and laser profiling equipment, was controlled via a Labview interface and communicated with the PC and robotic system through an RS232 serial interface. The execution module utilized a commercial industrial robot integrated with a pneumatically actuated shear end-effector. However, any unexpected carcass movement during scanning may increase the error rate of 3D mapping, and contact with the ground can lead to organ contamination. It is crucial to address these issues for brisket cutting in meat industrial production. Singh et al. (2012) reported another method for sheep brisket cut (Figure 2c). With this method, the problems in the above research have been improved to some extent. It obtains an offset of the carcass entry point by optical and ultrasonic sensors and calculates the cutting path based on the statistical method. The system accuracy would be limited by utilizing a fixed profile for a certain breed type, which may be incapable of detecting different types of carcasses. However, both of these brisket-cutting systems relied solely on visual inputs and lacked capabilities for sensing cutting forces. This made it difficult to adapt to changes during cutting, often leading to damage to internal organs due to the inability to adjust cutting forces in real time. Despite the process of precise brisket cutting is still extremely challenging, the above systems lay an essential foundation for future studies.

The automated systems for deboning specific livestock meat sections are still limited to laboratory settings, and it is still a long way from full automation. Thus, an alternative approach is establishing a human-machine collaboration platform and employing a robotic manipulator to assist with manual cutting to enhance efficiency and mitigate the risk of human injury (Sørensen et al., 1993). Longdell (1996) discussed various kinds of beef deboning machines for different sections of the animal, which are similar to primalisation pulling arms. Based on the above research, promoting deboning process robotization and intelligent systems is paramount to improving efficiency, scalability, flexibility, and sustainability. Research has been conducted on replacing the human hand with a robotic arm for deboning operations by learning from the butcher during processing (Figure 2d) (Essahbi et al., 2012; Friedrich et al., 2000; Guire et al., 2010a; Guire et al., 2010b; Zhou et al., 2009; Zhou et al., 2007). Wei et al. (2014) developed a dexterous robotic hand to replace the human operator’s hand in ham deboning (Figure 2e). The robotic hand comprises a re-configurable palm and four fingers to establish a hyper-flexible human-robot co-working platform in meat processing, including handing, pulling, pushing, and twisting. The four fingers perform abduction as well as flexion and extension, with adjustments made to the palm configuration for different tasks and changing environments. Motion trajectories of the operator’s left hand were captured via instrumented data gloves with appropriate force/torque and position sensors for mapping the deboning operation task workspace to the robotic hand joint space and performing human-robot co-working deboning operation. However, some critical issues such as reducing tendon-driven friction of the hand and increasing contact point friction between the meat and the robotic hand still need further investigation.

In recent years, there has been a focus on improving systems adaption ability, flexibility, and cost-affectation. To achieve precise cutting operations, current robotic brisket-cutting systems and deboning systems integrate multi-sensor feedback mechanisms to monitor cutting forces. However, the viscoelastic nature of meat tissues imposes stringent requirements on force-control accuracy during operation, significantly prolonging processing time. Furthermore, the presence of blood and other fluids in the cutting environment introduces additional challenges for sensor-based recognition, ultimately limiting the system’s precision. The ongoing development of artificial intelligence and machine learning is also expected to lead to further improvements in deboning automation technology in the coming years.

### 3.3 Commercial applications

Based on the technology mentioned above, there has been a growing popularity in recent times concerning commercial practices centered around the cutting of livestock (Xu et al., 2023).

Mayekawa Co. Ltd. from Japan has developed HAMDAS-RX, the world’s first automated ham-deboning robotic system with a maximum processing capacity of 500 hams per hour (Mayekawa, 2021). Upon the completion of pre-cutting processes, HAMDAS-RX can perform automated deboning of pork ham which includes effective extraction of hipbone and tailbone and differentiate between the right and left legs automatedly. Another company, SCOTT Automation Robotics Co. Ltd., employs a combination of robotic technology, scribing saws, and sensing technologies to accurately identify the position and shape of the beef (Scott, 2021). This design can reduce workloads of two to three per shift, and increase productivity remarkably.

The integration of advanced technologies, including robotics and sensing, has resulted in diminished reliance on manual labor, increased precision, and heightened productivity. Nonetheless, there is still a long way to go for the wide promotion of meat processing robots.

## 4 Poultry

In recent years, the escalating rise in the yearly production of poultry necessitated the implementation of robotized and mechanized approaches for the processing of poultry meat commodities (Elahi E et al., 2022). When compared to other meat processing industries, poultry processing is comparatively automated, except for certain challenging operations. Poultry processing consists of live-chicken stunning, slaughter, bloodletting, feathers removal, fluff removal, evisceration, trimming, pre-cooling, segmentation, and other processing operations (Casnor and Gavino, 2022; Janssen et al., 2007; Ramírez-Hernández et al., 2017). In Table 2, recent research on the identification and classification of poultry has been listed. In this section, we introduce the relevant techniques involved in poultry processing sequentially.

**TABLE 2:** Summary of technical progress in poultry evisceration.

References	Country	Technique	Hardware	Software and algorithms	Scenario	Performance	
Xiong et al. (2018)	China	Tactile perception	STM32	-	An experimental slaughtering machines	Residual rate	12.6%
Chen et al. (2020)	China	Machine vision	Industrial cameras	Morphological operation method Active contour algorithm	An experimental evisceration machines	Residual rate Breaking rate	6.2% 15%
Chen et al. (2020)	China	Machine vision	Industrial cameras	Active contour method	An experimental evisceration machines	Positioning accuracy	98.96%
Chen Y et al. (2023)	China	Machine vision	Industrial cameras	Active contour algorithms Image processing method	An experimental evisceration machines	Positioning accuracy	96.45%
Teimouri et al. (2018)	Iran	Machine vision	Charge-coupled device camera	Artificial neural network	An experimental evisceration machines	Overall accuracy	93%

### 4.1 Primary processing of poultry

The primary processing of poultry mainly includes de-feathering and evisceration. The procedure of de-feathering has been automated presently. In a friction-based process, dehairing and defeathering are typically achieved through a rotary rubber blade. Carcasses are subjected to plucking machines equipped with specially designed rubber “fingers” that effectively remove feathers.

**FIGURE 3:**
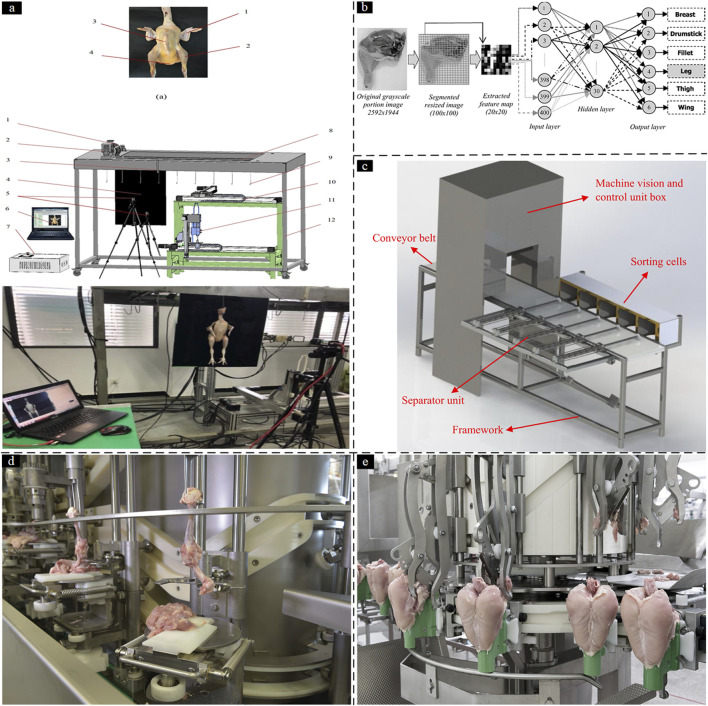
Poultry processing robot: **(a)** The acquisition system of the chicken evisceration (Chen et al., 2021b); **(b)** The poultry portion identification system: Image processing and neural network arbitration (Khashman, 2012); **(c)** The framework of chicken portion sorting machine simulated in CATIA software (Teimouri et al., 2018); **(d)** WLD Whole Leg Deboner M3.0 (Meyn, 1993). **(e)** RAPID plus breast deboner M4.3 (Meyn, 2023a).

The subsequent stage following defeathering is evisceration, which is a critical and challenging aspect of the process. As a result, manual labor is often relied upon to carry out this particular operation (Chen et al., 2021a). Currently, automated evisceration systems provide superior industrial processing capabilities when compared to manual evisceration (Li et al., 2021). Wang et al. (2018) proposed a system of poultry slaughtering robots based on a machine vision system, and then position the robot hand for grabbing the viscera using position recognition. The proposed control system incorporates dual operational modalities: manual and automated control configurations. In manual control mode, the robot arms and carcass conveyor can be controlled independently to facilitate kinematic calibration of the end-effector. In automated control mode, integrated with the vision-guided motion synchronization module, the system implements a closed-loop control architecture to maintain continuous and automated evisceration cycles. However, internal organs are easy to damage when machines perform consistent evisceration, most of them do not consider the internal organ’s integrity in the robotic grasping. Therefore, another machine-vision-based method was proposed by Chen and Wang (2018). In this method, evisceration can be executed by a multi-fingered robot hand mounted on the DELTA robot, which simulates a human hand and is more flexible than the previously designed manipulators. A threshold segmentation method is applied for poultry carcass recognition. The camera first takes poultry RGB (Red Green Blue) images on the conveyor, and then the images are processed by using a computer vision algorithm. The object positioning for internal organs can be calculated which indicates that the relative position is significantly changed between carcass and viscera with chicken size in the longitudinal direction (Figure 3a) (Chen et al., 2021a). Therefore, computer vision technology can be satisfactorily applied to predict the chicken viscera position.

### 4.2 Secondary processing of poultry

In the field of poultry processing, an additional significant concern pertains to secondary processing operations automation such as sorting, deboning, and meat quality evaluation. Different from other processing, manual sorting continues to be the most prevalent method used for sorting poultry portions before packaging. There are problems inherent with such manual sorting methods including high error rate, and worker fatigue (Nyalala et al., 2021). Based on the development of employing computer vision technology and ANN (Artificial Neural Network), Teimouri et al. (2018) proposed a new online method based on linear and nonlinear classifiers to categorize chicken portions automatically (Figure 3d). The geometrical features, color, and textural features were extracted from the image and selected by the Chi-Square technique, after which the classification was realized using ANN. This is the first attempt made to implement a system capable of sorting chicken portions in real-time practice using vision-based intelligent modeling.

As for the deboning process, there have been some solutions for deboning lines and cutting devices, but they still cannot automatically adjust to the variability of the poultry size (Daley et al., 1999; Heck, 2006; Guo and Lee, 2011; Woo et al., 2018). To solve this problem, Hu et al. (2012) designed an intelligent poultry shoulder deboning system by utilizing a knife equipped with a force sensor attached to a 2-DOF robot arm (Figure 3c). The system adopts a dynamic hybrid position/force control strategy. During the initial stage, position control is applied to track a predefined cutting trajectory. Upon detection of bone contact, the tool path is dynamically adjusted in real time to follow the tangential direction of the bone surface while maintaining a constant contact force to prevent bone chip formation. Similarly, Misimi et al. (2016) proposed a novel 3D vision-guided robot for front-half chicken harvesting. A computer vision algorithm has been developed to locate the grasping point in 3D as the initial contact point for the harvesting procedure, based on which, a feed-forward Look-and-Move control algorithm. A humanoid-inspired composite pneumatic gripper was employed as the end effector with a compliant design to adapt to the broiler chicken’s anatomy. A miniature force sensor ensures a precise grip and minimizes damage to the meat during handling. However, due to the restricted DOF, it is difficult to accomplish deboning of the entire carcass, and the force control will also be influenced by the shape of the blade. In the longer term, upgrading to a cutting robot possessing more DOF than the present could allow for more versatility in performing the various cuts required for complete poultry deboning. The two systems represent automated solutions for precision cutting and compliant grasping, respectively, collectively advancing the technological frontier of poultry processing automation.

Automation in the evisceration, breastbone deboning, grading, and packaging, has improved the overall production system in a fast and efficient manner. However, post-evisceration poultry inspection is still performed manually. The inspection system automation can eliminate inspector error and reduce the workload required for the carcass individual inspection, thereby reducing operating costs (Chao et al., 2000). Poultry meat color is also an important quality attribute for the rapid detection of “pale poultry syndrome”. Visual inspection is routinely used to assign grades or quality labels to chicken carcasses. Recently, novel techniques have been investigated for fast, reliable, and reagent-less meat quality assessment. For a fast assessment of chicken quality in large-scale processing plants, Barbin et al. (2016) investigated the potential application of an identification framework with color images to predict chicken color attributes and classify chicken breasts accordingly. This study is mainly concerned with detecting PSE (pale, soft, exudative) defects and pale poultry syndrome, which are critical indicators of pre-slaughter animal welfare and meat quality. While this work advances poultry quality assessment by enabling automated, high-throughput defect identification, further refinement is needed to ensure robustness and universal adoption across diverse production environments.

Current poultry processing automation demonstrates varying levels of technological maturity across different operations. While computer vision and ANN-based sorting (Teimouri et al., 2018) achieve real-time classification (with 90% accuracy), deboning automation remains constrained by limited DOF systems (Hu et al., 2012; Misimi et al., 2016), which struggle with anatomical variability and require force-vision hybrid control. In recent years, there has been a focus on improving system adaption, DOF, and reducing labor costs. These proposed strategies offer robust and promising alternatives for industrial poultry processing with high accuracy, rapid, and non-destructive methods.

### 4.3 Commercial applications

The poultry industry has built large, dedicated processing plants and continuously increased line speed through advancements in automation and mechanization of different processes within the plant (Barbut and Leishman, 2022).

Meyn Food Processing Technology B.V. Co. Ltd. (Meyn, 1993), the leading global manufacturer and marketer of systems and solutions for poultry and egg production, has proposed solutions to the entire poultry processing including live bird handling, slaughtering, evisceration, chilling, deboning, packing, and so on. The whole leg deboner M3.0 (Meyn, 1993) (Figure 3d) processes left and right anatomical legs at a maximum capacity of 4,200 legs per hour. Furthermore, the rapid plus breast deboner M4.3 (Meyn, 2023b) (Figure 3e) automates the breast deboning process by loading front halves in baskets, transferring them to product carriers, and then to a meat harvesting carousel and a carousel for wishbone cutting and scraping. This system can process both breast caps and front halves into over 15 different high-quality products at a speed of up to 7,000 BPH and saves up to 34 FTE per shift.

## 5 Seafood

Seafood products are essential dietary components with highly appreciated and consumed worldwide (Hassoun et al., 2022). The seafood’s perishable nature needs to be paid special attention to its preservation after harvesting. Automated seafood processing could enable higher profitability, and flexibility in production and increase the potential for high-value seafood products (Liu et al., 2022). The seafood industry has come a long way with automation advanced. Within this part, we present the pertinent methodologies implicated in the processing of seafood. These comprise categorization, slicing, the elimination of fish bones, and the evaluation of quality. Furthermore, specific commercial implementations that process seafood are also deliberated upon.

### 5.1 Primary processing of seafood

Sorting is considered an integral step in the primary processing of seafood, it can be categorized based on a factors combination of factors such as the species, size, and quality. In the early studies, fish was classified simply by its thickness (Booman et al., 1997). Later, various fish databases to automate the fish discrimination task were developed, and the application of machine vision and imaging technologies became increasingly prevalent in the sorting, grading, and processing of fish and its related products (Mathiassen et al., 2011; Hassoun et al., 2023).

With the rapid development of machine vision technology represented by CNN (Convolutional Neural Network), computer vision-based seafood detection and identification technology has entered a new stage of development. Compared to traditional vision-based algorithms, emerging machine vision algorithms have huge advantages in seafood recognition. In Table 3, recent research on the identification and classification of seafood has been listed. Utilizing edge analysis enables the identification and removal of malformed items while preserving those with growth potential. To develop more accurate systems for automated fish sorting based on whole-shape characters. Costa et al. (2013) presented an automated sorting for scale, sex, and skeletal anomalies of farmed seabass. The high-resolution camera is employed to capture lateral-view images of live fish. Image binarization is performed using both the grayscale (G) channel and the Value (V) channel from the HSV color space. The system is designed for integration with online sorting equipment, achieving a theoretical processing speed of approximately 10 fish per second. However, its real-time performance in dynamic industrial environments remains untested, which may affect operational stability in practical applications. This could be an important step forward both for the routine sorting of deformed fish at different stages and for the implementation of selective breeding programs through efficient selection based on body size and phenotypic sex.

**TABLE 3:** Summary of technical progress in seafood processing.

References	Country	Content	Technique	Software and algorithms	Performance	
Balaban MO et al. (2010)	U.S.A	Predict fish weight	Machine vision	Regression analysis	R2	0.987
Hernández-Ontiveros JM et al. (2018)	Mexico	Fish counting	Embedded system	A new algorithm for fish counting based on digital image processing	Accuracy	96.64%
Muñoz-Benavent P et al. (2018)	Spain	Highly accurate fish length estimation	Machine vision	Feature extraction algorithms	Estimation error	3%
Chen et al. (2021c)	China	Non-destructive freshness assessment	Machine vision	Hyperspectral imaging	Accuracy of fresh, refrigerated, and frozen samples	100% 96.43% 96.43%

**FIGURE 4:**
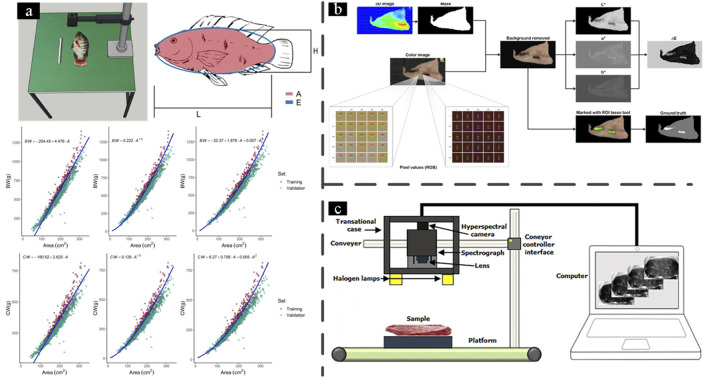
Seafood processing systems: **(a)** Representation of background removal, fish identification, and visual evaluation of goodness of fit for live body weight and carcass weight for models that considered only the segmented fish body area (Fernandes et al., 2020); **(b)** RGB and 3D images of an example fillet and the sequence of computer vision operations used to generate images, features, and the ground truth used for training of the classification algorithms. RGB pixel values of the normal muscle are higher (lighter color) than the pixel values of the blood spots (dark color) (Misimi et al., 2017); **(c)** Integration of hyperspectral imaging technology with spectroscopy and computing for quality evaluation of seafood products (Ismail et al., 2023).

In addition, fish weight estimation is also researched based on computer vision and image analysis (Figure 4a) (Fernandes et al., 2020; Zhang et al., 2020). There is also some research on counting systems (Hernández-Ontiveros et al., 2018) and sizing systems (Muñoz-Benavent et al., 2018). Moreover, the combination of computer vision procedure and acoustic information is expected to estimate biomass in more complex situations.

### 5.2 Secondary processing of seafood

There have been different methods for separate procedures in fish processing, such as fish beheading positioned with a cutting plane (Azarmdel et al., 2021), gutting beheaded and nonbeheaded fish were aspirated entrails and extracted blood and water from the abdominal cavity (Grosseholz and Neumann, 2008; Paulsohn et al., 2007), fish cutting and removal of the viscera without damage to either the viscera or the remaining fish product (Ryan, 2015).

In fish processing, the previous work on the integration of intelligent sensing, robots, and end effector tools for fish processing has resulted in several solutions based on machine vision and robots (Fu et al., 2023; Khodabandehloo, 2022; Mathiassen et al., 2011). Despite some singular unit operations having been successfully automated, and cooperatively with manual human labor, the current mechanical, semi-automated, and fixed automated solutions based on the existing technology are hardly able to perform a higher degree of automation in handling, processing, and higher raw material utilization. Based on the 3D vision system research, Mathiassen et al. (2011) established a salmon slaughter line by integrating a laser triangulation device with a robot to complete the salmon bleed-cutting task. The system first takes 3D images of fish in the production line and then completes the 3D segmentation of each fish. The fish head and tail are then graded according to the extracted characteristics. Finally, the entry point is set to direct the robot to complete the trimming process. This system can automatically slaughter 85-95 percent of all fish at an average feed rate of 30–80 salmon/min.

In today’s modern fish processing plants, the trimming operation is performed by a combination of automated trimming systems and manual post-trimming. Post-trimming includes the removal of belly fat, back fat, belly membrane, belly bone, collar bone, tail, blood, wounds, etc. Barbut and Leishman (2022) designed and implemented a prototype robotic post-trimming system for salmon fillets. The system integrated 3D machine vision, a high-speed robot manipulator, and a flexible lightweight cutting knife for enhanced tail-cut grounding. A smooth trajectory based on slow-motion analysis of human cutting motions, utilizing cubic Hermite interpolation for cutting path optimization. The six-degree-of-freedom (DOF) industrial robot is applied to enable flexible cutting paths for all relevant trimmed objects in a way that maximizes yield and minimizes waste.

There’s also some research focusing on blood spot detection. An image analysis method was developed to quantify the salmon fillets’ gaping, bruising, and blood spots (Balaban et al., 2011; Xu and Sun, 2018). An adaptive threshold value for lightness, depending on the fillet average color, was utilized to quantify the area with a luminance less than the threshold value under polarized light. However, this method cannot distinguish between gaping, bruising, and blood spots. With the development of more advanced image analysis technology, some deep learning algorithms like CNN and SVM (Support Vector Machine) were employed to realize a more robust classification approach (Misimi et al., 2017). Aligned RGB and depth images were used for image analysis as shown in Figure 4b.

As time passes, the quality of harvested fish may deteriorate due to storage and processing. As a result, evaluating the quality of processed fish has been extensively studied. HSI (Hyperspectral imaging) technology, a rapid and non-destructive tool, exhibits tremendous potential in evaluating the quality of seafood products through online or at-line detection (Ismail et al., 2023), thanks to its ability to provide spatial and spectral information coupled with multivariate analyses. As shown in Figure 4c, the HSI system is widely used in the seafood industry. Recently, artificial intelligence-based deep learning has emerged as a promising solution for data classification of hyperspectral imaging with shift-invariant feature recognition for seafood products. While complete automation remains challenging due to seafood’s biological variability, emerging technologies like hyperspectral imaging (Ismail et al., 2023) and deep learning (Misimi et al., 2017) now enable comprehensive quality assessment, surpassing traditional manual methods. These advances highlight the industry’s shift toward data-driven, high-throughput automation while ensuring product quality.

### 5.3 Commercial applications

Marel Co. Ltd. is the leading global supplier of advanced standalone equipment and integrated systems to the fish processing industry, which offers products of desliming, beheading, fillet processing, portioning, weighing, grading, and batching for salmon and whitefish.

The salmon fish is first measured to make the right cuts and ensure optimum yield. Then they are manually fixed in the gripper of the beheading machine MS 2730 (Marel, 2018) which can process up to 20 fish per minute. The salmon beheader is integrable with the filleter MS 2750 (Marel, 2023) with automatic transfer of the salmon straight from the deheader into the filleter with belly down. An additional set of circular knives cuts the fish from vent to tail. For the belly bone cut, four sets of finger pressures secure maximum control of the fish and enable optimum cutting of both pre-rigor and post-rigor fillets. The MS 2730 automatically adjusts to various fish sizes and can process up to 25 fish per minute depending on the length of the fish.

## 6 Discussion

Fresh meat is an abundant source of high-quality protein and essential trace minerals (Das et al., 2019). Nowadays, the demand for meat consumption has shifted towards personalized and diverse options that prioritize freshness, quality, and safety (Ren et al., 2022). Besides, there is a significant emphasis in the food industry on the unification of all supply chain processes (Barbut, 2020), particularly within the meat-producing sector, this involves incorporating data obtained through the monitoring of multiple stages involved in the processing plant, starting from the reception of live animals to the subsequent execution of various procedures. In recent times, the potential of robotics and automation as a promising method for meat processing has been thoroughly investigated. These technological advancements have unquestionably offered an advantageous foundation for establishing a traceability system within the meat production industry. To advance the robotization and automation of meat processing, it is imperative for future research to address the following issues.

Firstly, improve the software intelligence in the robotic system for meat processing. This includes boosting the efficiency of perception and recognition algorithms as well as the effectiveness of control systems to ensure the system functions optimally. In contrast to tasks performed by industrial robots, the operational environment encountered by meat processing robots is significantly more intricate and characterized by a greater degree of task uncertainty. Upon examining the historical progression of meat processing robots, and drawing a comparison between traditional vision techniques and CNN, it becomes clear that the iterative and methodical application of computer vision technology has substantially augmented the overall performance of these automated systems. Consequently, the implementation of inventive software algorithms remains a pivotal aspect in the continuing development of such robotics. Furthermore, sophisticated control strategies, like deep reinforcement learning, and imitation learning, may hold great potential for further enhancing the resilience and durability of robotic systems.

Secondly, achieve higher hardware performance of the robotic system, like designing nimble manipulations and enhancing the precision and robustness of sensors. Advanced meat processing techniques such as evisceration and deboning require robots with highly dexterous end-effectors. Recently, new actuator mechanisms that coordinate the movements of dual robotic arms have emerged. MRS (Multi-Robot Systems) presents many advantages over single robots, e.g., improved stability and payload capacity (Kennel-Maushart et al., 2022). There is potential for promising developments in meat processing robotics through the implementation of MRS and HRC (Human-Robot Collaboration). Thirdly, pay attention to the treatment of biological fertilizers generated in meat processing, and achieve sustainable production. During the process of slaughtering and meat processing, a significant amount of meat by-products and co-products are produced. These products need to be managed rationally to ensure ecological disposal (Irshad and Sharma, 2015; Toldrá et al., 2021). Therefore, it is critical to find efficient solutions that support sustainability. Innovative developments in this area can create high added value from meat by-products with minimal environmental impact, handling, and disposal costs, making it an essential component of the transition to bio-economy.

Additionally, automation enables the implementation of traceability systems, enhancing transparency across the meat supply chain. Leveraging big data analytics and IoT technologies can optimize resource efficiency and support agile food network systems (Lin et al., 2020). Such integration not only improves production monitoring but also advances research in food safety and quality control (Lin et al., 2020).

In summary, the core technological challenge in contemporary meat processing systems is balancing precision and throughput requirements. Even within the same species, substantial individual variations create significant obstacles for generic algorithms application. Current researches are increasingly focused on the 3D imaging modalities to obtain comprehensive carcass scans, theoretically enabling optimized cutting path planning. However, the high computational intensity prevents these systems from meeting industrial-scale processing rates. Additionally, The material properties of meat further complicate automation efforts. Meat as a non-uniform material, required precise control of cutting forces to maintain product integrity. While closed-loop control systems incorporating force feedback could potentially improve accuracy, the increased computational overhead and extended processing times decreased throughput. Consequently, existing meat processing systems mainly utilized the open-loop control strategy, sacrificing adaptability to individual variations in favor of operational efficiency through standardized cutting paths. This fundamental balance between processing accuracy and operational efficiency remains the central issue in advancing meat processing automation.

## 7 Conclusion

The meat processing industry presents significant opportunities for robotic automation, yet substantial challenges remain, particularly in livestock processing. This paper reviews the current status of robotic automation in meat processing. Full automation in livestock processing remains challenging, especially for beef due to its large size variations and complex structure, making a universal solution difficult to achieve. For poultry processing, there have been complete automation solutions to the whole slaughtering process, such as the products offered by Meyn Poultry Equipment Ltd. However, these current systems still have high requirements for consistency, and future research can focus on adaptability to variations in size. Fish processing is relatively simple due to its simple structure. Methods for deadheading, gutting, deboning, and filleting have been applied in the industry. The challenge is mainly in post-trimming, such as detecting various defects in fillets. Current research also focuses more on classification and quality control. With the development of computer vision techniques, the classification of species and sizes becomes more accurate which can enhance the yield of the production line.

Although the meat processing industry has great research value and development potential, it also faces many challenges. From the commercial and organizational aspects, the company needs to invest a great deal of cash in purchasing the equipment, which is not an easy task in the beginning. From the technical aspect, humans possess sophisticated, integrated sensory abilities with inbuilt reasoning and manipulation capabilities. It demands robotic and automated systems with highly accurate sensing systems and flexible processing strategies and methods.
